# Traumatic or Idiopathic Tetanus

**Published:** 1883-04

**Authors:** R. Lovell

**Affiliations:** Potsdam, New York


### II.—TRAUMATIC OR IDIOPATHIC TETANUS?

BY R. LOVELL, V. S.

A very valuable young stallion, æt. four, was put to five mares during the season. After this small amount of service he was kept in a box stall with a paddock adjoining, to which he had free access.

His diet during the rest of the summer consisted of a good quality of hay. The box stall, I might remark, was kept clean and in the best possible sanitary condition.

On Oct. 3rd this animal was taken to the smith's to be shod. The reason for having him shod was that he was to be trained during the winter. When he left the stable for the forge, there was no indication of disease of any kind.

The groom, however, informed me that "he made considerable fuss" while the smith was driving the nails in one of the forward feet; this resistance being so great that it was with difficulty that trainer and groom could render him submissive enough to have the shoe firmly secured.

This animal had been shod before without special annoyance to the workmen.

After being shod he was returned to the stable, and eat his hay that night and the following morning with an apparent relish.

At eight A. M. he was harnessed to a sulkey, and started for his training course. When started, the trainer found that he started with a "*straddling gait* as if *suddenly struck blind.*"

At this time I was called to see him, and on examination diagnosticated the case as one of tetanus.

I found the horse extremely nervous, massiter muscles tense and ridged, quivering of the whole body, breathing hurried, neck stiff jaws locked, saliva running from the mouth, and his breath very fetid. Membrane nicitans undisturbed, pulse running up at times to 80 per minute.

Treatment : Endeavored to give a cathartic :

℞

| | | |
|---|---|---|
| Aloes Barbadoes, | . . | ʒvi. |
| Hydrarg. Chloride Mitis, | . | ʒi. |
| G. Radix Po. | . . . | ʒii. |

Mix and put in drench.

When the above was ready it was impossible to get the horse to swallow. I then gave gr. iv. of sulphate of morphia every two hours to quiet the spasmodic twitching of the muscles. Also ext. belladonna ʒi every hour on the tongue. I also cleansed the rectum with the hand and gave an enema of soap and water every hour.

Also used the following liniment over the loins :

℞

| | | |
|---|---|---|
| Spts Camph., | . . . | ℥ii. |
| Lac Ammoniaca fort, et | | |
| Ol Sassafras, et | | |
| Chloroform, et | | |
| Spts. Terebinthinae, aa, | . . | ℥i. |
| Oleum Caryophylli, | . . | ℥ss. |
| Alcohol, | . . . . | ℥x. |

Mix sig. ex. use.

The animal was also warmly blanketed. He grew warm rapidly and about seven broke out into a profuse perspiration, but at eight P. M. he died.

POTSDAM, NEW YORK. *Jan'y*, 1883,

---

